# Risk Factors for Antimicrobial Use on Irish Pig Farms

**DOI:** 10.3390/ani11102828

**Published:** 2021-09-28

**Authors:** Lorcan O’Neill, Julia Adriana Calderón Díaz, Maria Rodrigues da Costa, Sinnead Oakes, Finola C. Leonard, Edgar García Manzanilla

**Affiliations:** 1 Pig Development Department, Teagasc, The Irish Food and Agriculture Authority, Moorepark, Fermoy, Co Cork P61 C996, Ireland; Julia.CalderonDiaz@teagasc.ie (J.A.C.D.); maria.costa@sruc.ac.uk (M.R.d.C.); egmanzanilla@gmail.com (E.G.M.); 2 School of Veterinary Medicine, University College Dublin, Belfield, Dublin 4 D04 W6F6, Ireland; nola.leonard@ucd.ie; 3 School of Public Health, University College Cork, Cork T12 K8AF, Ireland; sinnead.oakes@agriculture.gov.ie

**Keywords:** antimicrobial use, biosecurity, Ireland, pigs, respiratory disease, risk factors

## Abstract

**Simple Summary:**

Antimicrobial resistance (AMR) is a major threat to public health. There are concerns that antimicrobial use (AMU) in agriculture has a role in the development of AMR. Pigs are one of the main consumers of veterinary antimicrobials and a better understanding of the drivers for AMU in this sector will help in efforts to reduce use. The aim of this study was to investigate the associations between antimicrobial use, farm characteristics, biosecurity, the presence of respiratory disease on the farm and health management practices on Irish pig farms. Farms that manufactured their feed on-site had lower total AMU than farms that purchased their feed from a feed mill. Higher levels of lung abscesses and pericarditis (inflammation of the lining around the heart), both indicators of respiratory disease, were associated with increased AMU. Higher levels of pericarditis were also associated with increased use of critically important antimicrobials. Farms vaccinating against swine influenza also had higher AMU. Farms that administered prophylactic antimicrobial treatments to piglets had higher use of individual treatments and critically important antimicrobials. The results from this study show that prophylaxis and respiratory disease are the main drivers of AMU on Irish pig farms. These findings highlight areas of farm management where interventions may aid in reducing AMU on Irish pig farms.

**Abstract:**

The threat to public health posed by antimicrobial resistance in livestock production means that the pig sector is a particular focus for efforts to reduce antimicrobial use (AMU). This study sought to investigate the risk factors for AMU in Irish pig production. Antimicrobial use data were collected from 52 farrow-to-finish farms. The risk factors investigated were farm characteristics and performance, biosecurity practices, prevalence of pluck lesions at slaughter and serological status for four common respiratory pathogens and vaccination and prophylactic AMU practices. Linear regression models were used for quantitative AMU analysis and risk factors for specific AMU practices were investigated using logistic regression. Farms that milled their own feed had lower total AMU (*p* < 0.001), whereas higher finisher mortality (*p* = 0.043) and vaccinating for swine influenza (*p* < 0.001) increased AMU. Farms with higher prevalence of pericarditis (*p* = 0.037) and lung abscesses (*p* = 0.046) used more group treatments. Farms with higher prevalence of liver milk spot lesions (*p* = 0.018) and farms practising prophylactic AMU in piglets (*p* = 0.03) had higher numbers of individual treatments. Farms practising prophylactic AMU in piglets (*p* = 0.002) or sows (*p* = 0.062) had higher use of cephalosporins and fluoroquinolones. This study identified prophylactic use and respiratory disease as the main drivers for AMU in Irish pig production. These findings highlight areas of farm management where interventions may aid in reducing AMU on Irish pig farms.

## 1. Introduction

Antimicrobial resistance (AMR) is a continued and increasing threat to global public health [1]. Improved antimicrobial stewardship and ultimately reduced antimicrobial use (AMU) are key components of various action plans to mitigate this threat [2,3]. Antimicrobial use in livestock production is a focus of concern because it accounts for a large proportion of global AMU [4] and animals may act as a reservoir for AMR pathogens and AMR genes [5]. Furthermore, the literature suggests that restricting AMU in livestock can reduce AMR in animals and humans [6].

The pig sector is a major consumer of veterinary antimicrobials (AMs) and ranks highest in several countries [7,8,9]. In pig production, AMs are primarily administered as group treatments to piglets post-weaning, via oral routes of administration and more often at strategic times in the production cycle [10,11]. A better understanding of the risk factors for this AMU will aid in efforts to reduce it. Antimicrobials are used for the treatment, prevention and control of infectious disease [12,13,14], and respiratory and gastrointestinal disease are the major indications for use [11,15,16]. Therefore, it would be expected that disease burden is the primary driver for AMU. However, the relationship between disease burden and AMU is not well-characterised [17,18].

Farm characteristics and management practices such as herd size [19,20,21], proximity to other pig farms [20,22,23], raising finisher pigs on specialised farms (which may use multiple suppliers) [20,21], increased farrowing rhythm and longer suckling period [24] can also influence AMU. Furthermore, higher levels of biosecurity [24,25] and implementing some specific biosecurity practices, such as provision of changing facilities [26] and provision of boots to visitors [22], are also associated with reduced AMU. Vaccination is considered a key tool in disease control and in reducing reliance on AMU [27,28,29,30]. However, despite effectiveness in field studies [31,32,33], vaccination has been associated with increased AMU in several studies [17,24,30,34,35]. Finally, socio-economic and demographic factors such as age, gender, education or years of experience [36] and attitudes to AMR [37] can influence on farm AMU, although their relative importance appears to differ depending on nationality [17]. In general, pig farmers perceive AMs as an effective and cost-efficient tool in disease management [30,38,39,40] and, at least in the past, were poorly aware or unconcerned about the risks of AMR [38,39,41].

There is little information about the factors associated with the choice of AM treatment, i.e., active ingredient and route of administration, on pig farms [42]. The various AMs have specific indications for use, and many are licensed to treat more than one condition. There are official national guidelines dictating which AMs should be used for specific conditions [43,44]. However, they are not always followed [15,45] and there can be notable variation in choice of treatment for the same condition [46]. A better understanding of the reasons behind such choices may aid in improving antimicrobial stewardship and would be especially useful for the highest priority critically important antimicrobials (HP CIA) which are most important to public health.

According to Collineau et al., the relative importance of risk factors associated with AMU varies between countries [17]. In Ireland, pig production accounts for approximately 40% of veterinary AM consumption, with prophylactic administration of medicated feed being the dominant practice [47], but the drivers for this use on Irish farms have not been studied previously. This study aimed to explore the risk factors for AMU in a cohort of Irish farrow-to-finish farms using AMU, biosecurity, respiratory disease and farm management data. A further aim was to investigate risk factors for specific AMU practices, namely the choice of AM class and route of administration.

## 2. Materials and Methods

### 2.1. Farm Selection

This cross-sectional study was conducted on a sample of 52 Irish pig farms to investigate risk factors for AMU. The risk factors investigated were grouped as follows: biosecurity practices; farm management practices, including vaccination; farm status and prevalence of respiratory disease; and farm performance indicators. The study farms were part of a larger cohort of farms that participated in two separate but closely related projects, investigating (1) antimicrobial use and (2) respiratory disease, which were conducted from February 2016 to September 2018. All participants were clients of the Teagasc (Teagasc, the Agriculture and Food Development Authority, https://www.teagasc.ie/animals/pigs/ accessed on 20 September 2021) farm advisory service, which is available to all Irish pig farms. In 2017, the Teagasc farm advisory service included 107 pig farms, representing over 77,000 sows (approximately 50% of the national herd) [48]. All client farms were invited to participate in both projects on a voluntary basis, and the 52 farms in this study represent those that provided sufficient data to both the AMU and the respiratory disease projects.

### 2.2. Farm Production and Performance

The study farms submit their production data to the Teagasc e-Profit Monitor (ePM) database quarterly. The following indicators for the 2016 calendar year were extracted from the ePM database: herd size, piglet, weaner and finisher mortality, average age at weaning, at transfer to finisher and at slaughter (which were used to calculate length of stay in the weaner and finisher stages), average daily gain (ADG) and feed conversion ratio (FCR).

### 2.3. Antimicrobial Use

Antimicrobial use data for the 2016 calendar year were collected for each farm by means of farm visits conducted from September 2017 to September 2018, as previously described by O’Neill et al. [47]. In short, details of AMU in medicated feed regarding the diets and age groups treated and the AMs used were provided by the farmers. The amounts of AMs in oral remedies other than premix (i.e., not for medicated feed) and injectable preparations were determined using invoice and or prescription records. Feed consumption data (to calculate amount of medicated feed) and population data were extracted from the ePM database. Treatment incidence (TI), which represents the percentage of pigs in a stage of production treated with a dose of AM each day (or equivalently, the percentage time of the period at risk for which a pig was treated) [49], was calculated for each AM and route of administration in each stage of production (piglet, weaner and finisher). The TI was calculated using the formula adapted by Sarrazin et al. [11] and the Defined Daily Dose (DDD_vet_) for each AM, as assigned by the European Medicines Agency (EMA) [50], and standard weights for each age group, as proposed by the European Surveillance of Veterinary Antimicrobial Consumption (ESVAC) project [51]:

(1) $${\mathrm{TI}}_{\mathrm{DDDvet}}=\frac{\mathrm{amount}\;\mathrm{of}\;\mathrm{antimicrobial}\;\mathrm{used}\;\left(\mathrm{mg}\right)}{{\mathrm{DDD}}_{\mathrm{vet}\;}\;\left(\mathrm{mg}/\mathrm{kg}\right)\times \mathrm{kg}\;\mathrm{of}\;\mathrm{animal}\;\mathrm{at}\;\mathrm{risk}\;\left(\mathrm{kg}\right)\times \mathrm{number}\;\mathrm{of}\;\mathrm{days}\;\mathrm{at}\;\mathrm{risk}}\times 100\;\mathrm{animals}\;\mathrm{at}\;\mathrm{risk}$$

The TI values for piglets, weaners and finishers were then combined to calculate the standardised TI200 indicator, which represents AMU for the entire rearing period, using the formula defined by Sjölund et al. [10]:

(2) $$\mathrm{TI}200_{\;}=\frac{{\mathrm{TI}}_{\mathrm{piglet}}\;\times \mathrm{suckling}\;\mathrm{period}+{\mathrm{TI}}_{\mathrm{weaner}}\times \mathrm{weaner}\;\mathrm{period}\;+{\mathrm{TI}}_{\mathrm{finisher}}\times \mathrm{fininshing}\;\mathrm{period}}{\mathrm{total}\;\mathrm{rearing}\;\mathrm{period}}\;\times \frac{200\;\left(\mathrm{standard}\;\mathrm{life}\;\mathrm{span}\right)}{\mathrm{total}\;\mathrm{rearing}\;\mathrm{period}}$$

Information on which vaccinations were used on the farm and whether the farm administered prophylactic AM treatments (other than in medicated feed) in the different production stages (by any route of administration) was also collected for each farm.

### 2.4. Biosecurity Assessment and Farm Management Practices

Biosecurity practices were assessed using the Biocheck.UGent^TM^ (https://biocheck.ugent.be/en accessed on 20 September 2021) questionnaire, which was completed during the course of farm visits conducted between February and May 2016 [52]. The Biocheck.UGent^TM^ questionnaire consists of 109 closed questions which assess various biosecurity measures and farm management practices. Questions on farm characteristics do not contribute to the biosecurity score and include the age of the oldest and youngest buildings, the number of employees and the experience of the farm manager/owner. Information on whether the farm milled its own feed or purchased from an external feed mill was also collected. Biosecurity-related questions are grouped into six external biosecurity categories and six internal biosecurity categories. Each question contributes a weighted score to its category and each category contributes a weighted score to the external or internal biosecurity score. The overall biosecurity score is computed as the average of the external and internal biosecurity scores. Scores range from 0 to 100, where 0 indicates the absence of biosecurity and 100 represents perfect biosecurity. The questionnaire for pigs is described in detail by Laanen et al. [25]. The scores for each category as well as the internal, external and overall biosecurity scores were used in the statistical analysis.

### 2.5. Respiratory Disease Status and Farm Prevalence

Data on disease status and prevalence of pluck (lungs, heart and liver) lesions were collected from November 2017 to April 2018, as previously described by Rodrigues da Costa et al. [53]. Disease status for Influenza A virus (IAv), Porcine reproductive and respiratory syndrome virus (PRRSv), *Mycoplasma hyopneumoniae* (Mhyo) and *Actinobacillus pleuropneumoniae* (APP) was determined using serological testing. Thirty-two samples per farm were collected and processed at the Blood Testing Laboratory of the Department of Food, Agriculture and the Marine (Cork, Ireland). Serum was separated from the samples and stored at −80 °C until further analysis. For analysis, all 32 samples were used for detection of IAv and PRRSv (allowing for a minimum within-herd prevalence of 10%, α = 0.05), while 16 samples were used for detection of Mhyo and APP (allowing for a minimum within-herd prevalence of 18%, α = 0.05). The seroprevalence of antibodies against IAv, PRRSv, Mhyo and APP were determined using the respective IDEXX ELISA kits, for the four respiratory pathogens: Influenza A Ab Test, PRRS X3 Ab Test, HerdChek *Mycoplasma hyopneumoniae* Ab Test and APP-ApxIV Ab Test (IDEXX, Hoofddorp, The Netherlands), according to the manufacturer’s instructions. Farms were considered positive if at least one sample tested positive by the relevant serological test.

All pluck examinations were carried out by a single trained veterinarian. Lungs were removed from the carcass by abattoir personnel and scored in the evisceration line for pneumonic lesions using the method described by Madec and Derrien [54]. Pleurisy in the dorsocaudal lobes was scored using a modified version of the Slaughterhouse Pleurisy Evaluation System (SPES) [55,56] with a 4-point scale, in which 0 = no pleurisy, 2 = focal lesions in one lobe, 3 = bilateral adhesions or unilateral lesions affecting more than 1/3 of one diaphragmatic lobe and 4 = extensive lesions affecting more than 1/3 of both diaphragmatic lobes (SPES score = 1 corresponds to cranial pleurisy and is not used in the modified SPES scoring system). The prevalence of dorsocaudal pleurisy, i.e., lesions with SPES score ≥ 2, and the prevalence of moderate or severe dorsocaudal pleurisy, i.e., lesions with SPES score 3 or 4, were used for statistical analysis. Additionally, scars, indicative of healing pneumonic lesions from previous infection, cranial pleurisy (adhesions between lobes, on the surface of the apical and cardiac lobe, and/or adhesions between the lung and the heart), pericarditis (expansion of the pericardial cavity with inflammatory exudate [57]), lung abscesses and liver spots (indicative of transhepatic migration of the larvae of *Ascaris suum* [58]) were recorded as present or absent.

### 2.6. Data Processing and Statistical Analysis

All data were entered into a Microsoft^®^ Excel 365 (Microsoft Corporation, Redmond, WA, USA) spreadsheet. Calculations and statistical analyses were carried out using Microsoft^®^ Excel and R version 3.4.2 [59]. Data visualisation was carried out using the ggplot2 and VennDiagram packages in R [60,61]. Two separate analyses were carried out to investigate risk factors associated with (1) quantitative AMU and (2) AMU practices.

For the quantitative AMU analysis, the outcome variables were total TI200 for group oral treatments administered (TI_group_), total TI200 for individual treatments (TI_individual_), combined TI200 for ceftiofur and fluoroquinolones (TI_ceflq_) and total TI200 for all routes of administration (TI_total_). Variables were checked for normality by examining the quartile-quartile plot and by using the Shapiro–Wilks test. The four outcome variables were not normally distributed and thus, a log transformation was applied to approach a normal distribution. The transformation of TI_group_ and TI_ceflq_ required the addition of a constant (1 and 0.01, respectively) to account for farms with zero AMU in these categories. Collinearity among the predictor variables was checked using Spearman rank correlations for continuous variables, Wilcoxon rank sum test for continuous and categorical variables and chi squared or Fisher’s exact test for categorical variables. Categorical predictor variables (all were binary in this dataset) with less than 10 farms in either category were excluded from further analysis. For each outcome variable, the associations with each of the predictor variables were first assessed using univariable linear regression models. A multivariable linear regression model was then created using predictor variables with *p* ≤ 0.25 in the univariate analysis. Where there was collinearity among the predictor variables (Spearman rho > 0.7 or *p*-value of chi-square/Fisher tests ≤ 0.05), the variable with the strongest association in the univariable model or the one with the most biological significance was selected. Alpha level for determination of significance was 0.05 and trends are discussed between 0.05 and 0.1. The model was then refined using manual backward selection, removing the predictor variable with the highest *p*-value until all remaining variables had a *p* ≤ 0.1.

Associations between specific AMU practices and the predictor variables were investigated using logistic regression. Here, the outcome variables were each combination of AM class and the two main routes of administration: oral group treatments (eight outcome variables) and individual treatments (eight outcome variables). Collinearity among the predictor variables was checked as per the quantitative AMU analysis and categorical variables with less than 10 farms in either category were excluded from the analysis. Univariable associations between each outcome variable and predictor variable were assessed using chi-squared or Fisher’s exact test along with the respective odds ratio if the predictor variable was categorical, and univariable logistic regression for continuous variables. Thresholds for significance and tendency were set as for the linear regression analysis. Multivariable logistic regression models were then created using predictor variables with *p* ≤ 0.25 in the univariate analysis and refined using manual backward selection until all remaining variables had a *p* ≤ 0.1.

## 3. Results

A summary of the farm characteristics and production data is presented in Table S1. The 52 farms had a combined population of 38,764 sows and thus represented approximately 29% of the national herd in 2016. The median herd size was 650 sows (range: 113–2354) and all farms operated a farrow-to-finish system which accounts for virtually all pig production in Ireland [62]. Twenty-two farms (42.3% of sample) practised home milling, whereby at least one diet was manufactured on the farm, while the remaining farms purchased all feed. Antimicrobial use expressed as TI200 is summarised in Table 1. The median TI200 was 14.2 (range: 0.2–169.1). Oral group treatments accounted for 93.3% of consumption (81.5% via medicated feed, 11.6% via water) and were administered on all but two farms. Provision of medicated feed to piglets via starter and/or link diets during the first 7 to 21 days post-weaning was standard practice on 44 farms (84.6% of sample), and 27 farms (51.9% of sample) provided medicated feed in the subsequent diets (see O’Neill et al. [47] for a full description). All farms administered individual AM treatments.

**Figure 1:**
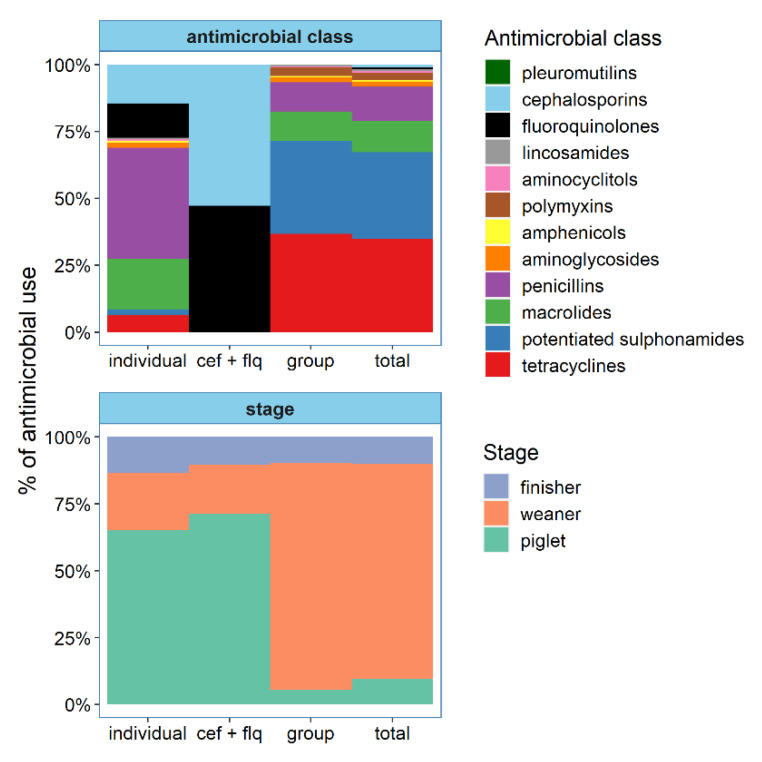
Breakdown of antimicrobial use by antimicrobial class and stage of production for individual treatments, cephalosporin and fluoroquinolone treatments, group oral treatments and total antimicrobial use on 52 Irish farrow-to-finish farms during 2016. Percentage of use refers to the contribution of the antimicrobial class or stage of production to the overall treatment incidence (TI200) indicator. Legend: cef + flq—use of cephalosporins and fluoroquinolones.

The breakdown of AMU by AM class and stage of production is shown in Figure 1. The most frequently used AM classes overall were tetracyclines, potentiated sulphonamides, penicillins and macrolides, accounting for 34.8%, 32.5%, 12.9% and 11.5% of AMU, respectively. Fluoroquinolones and cephalosporins accounted for 1.8% of AMU and were used on 90.4% of farms. Weaner pigs received 80.5% of all doses, but piglets accounted for 65% of individual treatments. The biosecurity scores for the study farms are summarised in Table S2. Farms typically had higher scores in external biosecurity (median 79.5 (range: 62–94)) than in internal biosecurity (60 (range: 29–80)). The category with the highest score was ‘purchase of animals and semen’, where 49 farms (94.2% of sample) had a perfect score, while the lowest scoring category was ‘cleaning and disinfection’ (median 38 (range: 0–95)). The farm vaccination and AM prophylaxis practices are summarised in Table S3. All farms administered *Erysipelothrix rhusopathiae* and parvovirus vaccination to sows. Vaccination against IAv and PRRSv was carried out on 19 and 21 farms, respectively (of which 11 vaccinated against both). Piglets were vaccinated against porcine circovirus type 2 (PCV-2) on 94.2% of farms and 75% also vaccinated against Mhyo. Half of all farms administered prophylactic AM treatments to piglets, typically in the first week of life either after birth or at processing (teeth clipping, iron injection, tail docking, etc. (castration is not routine practice in Irish pig production)). Twenty farms (38.5% of sample) administered prophylactic treatments to sows mainly via medicated feed or injections around farrowing or weaning. A summary of farm disease status for IAv, PRRSv, Mhyo and APP, and the prevalence of pluck lesions at slaughter, are shown in Table S4. All but one farm was positive for APP. Co-infection with other pathogens was common: 80.7% of farms (*n* = 42) were positive for at least three of the four pathogens, while 38.4% (*n* = 20) were positive for all four (see Figure S1).

The univariable linear regression models for the TI_total_, TI_group_, TI_individual_ and TI_ceflq_ are summarised in Table S5. The final multivariable models are shown in Table 2. There were significant positive associations between all the pleurisy-related variables (*p* ≤ 0.05) and both TI_total_ and TI_group_, but these were not retained in the final models. The final multivariable models for TI_total_ and TI_group_ were similar and explained approximately 53% and 55% of the variability, respectively. Farms with higher mortality in the finisher stage and those vaccinating against IAv had higher AMU, while farms home milling and farms with longer weaner stages had lower AMU (*p* ≤ 0.05). There were positive associations between prevalence of lung abscesses and pericarditis and AMU, although these were not significant for the TI_total_ (*p* ≤ 0.1). The final multivariable model for TI_individual_ explained 29% of the variability, with the administration of prophylactic AM treatments to piglets, higher biosecurity score in ‘vermin and bird control’ and prevalence of liver milk spots being positively associated with individual AMU (*p* ≤ 0.05). Farms with better biosecurity scores in ‘feed, water and equipment supply’ had reduced individual AMU (*p* = 0.007). Regarding the use of fluoroquinolones and third-generation cephalosporins, the model for TI_ceflq_ explained 37% of the variability. Farms with prophylactic AMU in piglets, those with lower biosecurity scores in ‘feed and water supply’ and those with higher prevalence of pericarditis used more of these classes (*p* ≤ 0.05). Farms with higher weaner mortality used less (*p* = 0.002), while farms which administered prophylactic AM treatments to sows tended to use more (*p* = 0.062).

The univariable associations between the predictor variables and the use (or not) of the various combinations of antimicrobial class and route of administration (group or individual) are shown in Table S6. The results from the final logistic regression models for AM classes used as group oral treatments are shown in Table 3 and Table S7 and are presented as odds ratios with their 95% confidence intervals. The associations identified in the quantitative analysis of total and group oral AMU were also identified as risk factors for use of the most consumed AM classes. Home milling farms were less likely to use tetracyclines (*p* = 0.048) and farms with a longer weaner stage were less likely to use tetracyclines, potentiated sulphonamides or lincosamides as group treatments (*p* < 0.05). Higher finisher mortality was associated with increased odds of potentiated sulphonamide use (*p* = 0.033) and macrolide use (*p* = 0.014). Farms positive for IAv were less likely to use oral penicillins (*p* = 0.041), but those vaccinating against IAv were more likely to use oral tetracyclines, potentiated sulphonamides and macrolides (*p* < 0.05). Higher prevalence of pericarditis was associated with increased odds of penicillin use (*p* = 0.007) and polymyxin use (*p* = 0.046). Farms that administered prophylactic AM treatments to piglets were 10 times more likely to use oral penicillins (*p* = 0.006). Higher scores in the biosecurity categories ‘nursery unit management’ and ‘measures between compartments’ were associated with decreased odds of tetracycline use (*p* = 0.04) and polymyxin use (*p* = 0.026), respectively. Higher scores in the ‘feed, water and supply of equipment’ and ‘environment and region’ categories tended to reduce the odds of potentiated sulphonamide use (*p* < 0.1).

The results from the final logistic regression models for AM classes used as individual treatments are summarised in Table 4 and Supplementary Table S8. All farms used injectable penicillins (41.7% of all individual treatments), and thus this class was excluded from the analysis. There was a tendency for increased odds of injectable tetracycline use on farms with higher weaner mortality (*p* = 0.072). Farms with higher piglet or finisher mortality tended to be less likely to use injectable macrolides (*p* < 0.1). The number of sows per employee was positively associated with the use of injectable aminoglycosides and third-generation cephalosporins (*p* < 0.05). Higher scores in the biosecurity categories ‘disease management’ and ‘feed, water and supply of equipment’ were associated with decreased odds of tetracycline use (*p* = 0.08) and fluoroquinolone use (*p* = 0.028), respectively. However, a number of biosecurity categories were associated with increased odds of use for certain AM classes. Farms with higher prevalence of liver milk spot lesions were less likely to use tetracyclines (*p* = 0.021) but more likely to use lincosamides (*p* = 0.017). In agreement with the findings for the TI_ceflq_ model, farms that administered prophylactic AM treatments to piglets were 4 times more likely to use third-generation cephalosporins (*p* = 0.074), and those that administered prophylactic AMs to sows were 17 times more likely to use fluoroquinolones (*p* = 0.088).

## 4. Discussion

This study explored the risk factors for AMU on Irish pig farms using data collected during previously published studies investigating AMU, biosecurity and respiratory disease on Irish pig farms [47,52,53]. The study farms were clients of the Teagasc advisory service and participated on a voluntary basis. This may have introduced selection bias, where farms with a prior interest in improving herd health were more likely to enrol, and therefore it cannot be assumed that the study sample is representative of the entire industry. Nevertheless, it represented approximately 29% of the national herd, and the findings of this investigation provide useful insights into the drivers for AMU in the commercial pig sector in Ireland. The linear regression models identified the main risk factors for the amounts of AMs used overall and for the primary modes of administration (group oral and individual), as well as for the fluoroquinolone and third-generation cephalosporin classes. The latter category is of interest due to the importance of these classes to public health and concerns over resistance [63,64], as well as the fact that, although volumes are relatively low, use is observed on most Irish farms [47]. The models for all AMU (TI_total_) and oral group treatments (TI_group_) provided similar results because the majority of treatments are administered orally, particularly via medicated feed. Individual AMU and cephalosporin/fluoroquinolone use also had risk factors in common, which is unsurprising since cephalosporins (injectable) and fluoroquinolones (injectable or oral dose) are relatively common individual treatments. The logistic regression models identified a number of risk factors for the use of certain combinations of administration route and class of AM. It should be noted that these models did not distinguish between low or high use, for example, the absence of use of a particular AM does not imply less AMU overall as another class may be used in its stead. For ease of interpretation, the results are discussed below in terms of the broad categories of risk factors.

### 4.1. Farm Characteristics

Farms home milling at least one of their diets had approximately 66% lower AMU than those that purchased all their feed. In particular, they were less likely to use group tetracycline treatment (*p* = 0.048), which was the most widely and heavily used oral treatment. This may relate to structural differences in AM delivery as home milling farms are not permitted to manufacture medicated feed unless they also possess a licence to do so [65]. Therefore, while home milling farms still have access to AMs, the “ease” of access differs between both groups, and this may point to a social factor whereby convenience and habit could explain some of the routine prophylactic AMU in medicated feed practised on the study farms. On the other hand, home milling may confer health benefits as they are reported to have lower finisher mortality compared to those purchasing their feed [66]. The reasons for this are not clear but could relate to the diet form, for example, lower prevalence of *Brachyspira piloscoli* was reported for Danish farms that provided home-milled or non-pelleted feed [67].

Older weaning age has been associated with lower AMU on European farms [17,24], likely because older piglets are less susceptible to the associated stresses of moving, mixing and diet change [68,69]. In this study, farms with a later weaning age were more likely to use macrolide group treatments (*p* = 0.037), but weaning age was not associated with the quantity of AMU. The reason for the association with macrolide use is not obvious, but the lack of association with quantitative AMU is likely due to insufficient variability between farms as most of them weaned pigs at 29 days. After weaning, the weaner stage on Irish farms is typically split into two sections, roughly 4–5 weeks each, with different housing and feeding infrastructure. Medicating throughout both phases was common on the studied farms. Contrary to the authors’ belief that a longer weaner phase might be associated with increased AMU, farms with a longer stay in the weaner stage had lower AMU. These farms were less likely to use oral tetracyclines or potentiated sulphonamides, the classes that contributed most to overall use, and they were also less likely to use oral lincosamides. The contrary results could relate to differences in accommodation associated with a longer weaning phase.

Farms with a higher number of sows per employee were more likely to use polymyxins (colistin) (*p* = 0.072) and cephalosporins (ceftiofur) (*p* = 0.029). Conversely, such farms were less likely to use oral aminoglycosides (apramycin) (*p* = 0.035). Colistin and apramycin are primarily indicated for gastrointestinal disease [14], and the conflicting results here may relate to the choice of one over the other on these farms when treating gastrointestinal disease. Colistin and ceftiofur are both HP CIAs, and the observation that less well-staffed farms are more likely to use them warrants closer inspection. These farms may have more health problems due to a lack of staff and thus may be more inclined to use so-called ‘last resort’ drugs. Convenience may be a factor too as the most widely used formulation of ceftiofur is long-acting, although this association was not observed for other long-acting parenteral AMs (e.g., macrolides).

### 4.2. Farm Performance Indicators

Finisher mortality is impacted by a variety of factors, including management, environment and infectious disease [70,71], and in this study, each additional 1% increase in mortality was associated with 33% higher AMU. This association has been previously demonstrated for the use of in-feed medication in Spain and the UK [26,72] and for AMU on heavy pig-fattening farms in Italy [73]. Farms with higher finisher mortality were also more likely to use oral potentiated sulphonamides or macrolides. Given that tetracyclines were the most applied group treatments, this might indicate a greater likelihood to deviate from the ‘standard’ use of tetracyclines. Since finishers accounted for just 10% of total AMU, finisher mortality may also reflect overall health through all stages of production. In this study, all farms that used medicated feed in finisher pigs also provided medicated diets in the weaner stage, suggesting that these farms may also have health problems in the earlier stages. Higher weaner mortality was associated with lower use of fluoroquinolones and cephalosporins (TI_ceflq_). It might be expected that weaner mortality would increase the use of these drugs, but farms with higher weaner mortality due to infectious disease may be more likely to focus on group treatments and, in this study, tended to have higher odds of parenteral tetracycline use (*p* = 0.072).

### 4.3. Biosecurity Practices

In contrast to other European studies, there was no association between the internal, external or overall biosecurity scores and total AMU [24,25]. Irish farms generally had high external biosecurity scores, mainly because most farms are closed herds, neither buying replacement gilts or finisher pigs from other sources. This may partly explain the lack of association with AMU. However, farms with better scores in the biosecurity category of ‘feed, water and equipment supply’ had lower use of individual treatments including cephalosporins and fluoroquinolones, and they were also less likely to use oral potentiated sulphonamides. Feed and water as well as their associated delivery and storage are potential sources of pathogens [74]. There were other associations between certain biosecurity categories and patterns of use. Better scores in the biosecurity category ‘measures between compartments’ were associated with lower odds of using polymyxins, which may link poor biosecurity with gastrointestinal disease. Although biosecurity scores were not associated with overall AMU in this study, there is evidence that farms which improve their biosecurity can reduce their AMU [75,76]. The generally lower internal biosecurity scores on Irish farms [52] demonstrate an opportunity for improvement, and the PigHealthCheck initiative run by Animal Health Ireland, which provides for free biosecurity assessments, is helping to raise awareness on this topic (https://animalhealthireland.ie/programmes/pig-healthcheck/introduction/ last accessed 20 September 2021).

### 4.4. Respiratory Disease

Respiratory disease is an important cause of mortality in pig production [77] and a major indication for AMU. In this study, neither farm status for IAv, PRRSv, Mhyo or APP, nor the prevalence of pneumonia or pleurisy were directly associated with total or group AMU. Instead, pericarditis and lung abscesses were positively associated with AMU, which increased by 7% and 6% respectively, for each percentage increase in farm prevalence. Pericarditis was also associated with higher fluoroquinolone use (approximately 15%) and higher odds of oral penicillin use (*p* = 0.007). Fluoroquinolones and penicillins are both are indicated for the treatment of APP infection and the latter is often used to treat and control *Streptococcus suis* infection [14]. Lung abscesses and pericarditis may be extensions of pneumonia and/or pleurisy involving infection with APP, *Pasteurella multocida* or *Streptococcus suis* [78], or they may be sequelae of systemic infections such as *S. suis* or *Glaesserella parasuis*. Thus, these lesions may be indicators of general health on the farm as *S. suis* and *G. parasuis* are also implicated in meningitis, polyserositis and arthritis, which are common conditions in pigs post-weaning [79,80]. Higher individual AMU associated with increased prevalence of liver milk spots at slaughter may be a direct result of *Ascaris suum* infestation, or it may indicate deficiencies in farm hygiene. *Ascaris suum* infestation has also been shown to interfere with Mhyo vaccination [81], further highlighting the importance of parasite control on pig farms.

### 4.5. Disease Management

Farms that administered prophylactic AM treatments to piglets had approximately 82% higher individual AMU (TI_individual_), higher use of cephalosporins and/or fluoroquinolones (TI_ceflq_) and they were also 10 times more likely to use oral penicillins (*p* = 0.006). Prophylactic AMU in piglets was observed on 50% of the study farms and is also common in other countries [82,83]. It usually occurs in the first week of life alongside procedures such as teeth clipping, tail docking and iron injection. The purpose of these treatments is to prevent infections associated with these procedures or with neonatal diseases endemic to the farm (e.g., meningitis or colibacillosis). Amoxicillin injection was the most common treatment used, but ceftiofur, enrofloxacin and long-acting macrolides were also used. The association with oral penicillin use might indicate farms using both strategies to control *S. suis* infection. Whether these treatments reduce morbidity and mortality on these farms is unknown; however, this finding shows that the practice does not reduce AMU. Farms that administered prophylactic treatments to sows tended to use more fluoroquinolones in growing pigs. This may reflect the disease status on certain farms or perhaps a behavioural pattern, where injudicious AMU practices co-occur on some farms (or it may reflect the corollary: good antimicrobial stewardship on some farms neither practising prophylaxis nor using HP CIAs). Farms vaccinating against IAv had 2.9 times higher TI_total_ compared to non-vaccinating farms. A similar association was observed in French herds in the pan-European study carried out by Collineau et al., who also reported other positive associations between vaccination and AMU for a number of country/pathogen combinations [17]. In Denmark, a retrospective study investigating trends in AMU and vaccination between 2007 and 2013 found no link between increased vaccination and reduction in AMU that occurred during this period [84], while others found that vaccination was associated with higher AMU [34,35]. This may seem counter-intuitive, but the authors of these studies suggest that farmers may use vaccinations to control diseases that are already on the farm in conjunction with AMs, while conversely, farms free from a given disease do not need to vaccinate and may not need AMs. In the present study, higher AMU on farms vaccinating against IAv may be a consequence of more severe respiratory disease. An association between influenza and pleurisy was reported for the larger cohort of Irish farms [53] and by others [85]. Co-infection is a common feature of the Porcine Respiratory Disease Complex (PRDC), and often, these co-infections are synergistic [86]. It is noteworthy that 11 of the 19 farms vaccinating against IAv also vaccinated against PRRSv. This is an interesting observation given that Calderon Diaz et al. found that Irish farms vaccinating against IAv or PRRSv are less profitable than farms that were positive but not vaccinating [87]. This could suggest that farms with high AMU are also less profitable, but further research is required. These findings highlight that vaccination alone may not be enough to eliminate disease and reduce AMU. However, their proven efficacy against a range of porcine infectious diseases [31,32,33] means that they are a key tool in this regard [28].

### 4.6. Implications for Irish Pig Sector

Prophylaxis was one of the main drivers of AMU in this study. This was seen in the relationship between AMU and home milling and in the influence that prophylactic practices in sows and piglets had on the use of oral penicillins and HP CIAs. European Union regulations from 2022 mean that prophylactic AMU in animals will be banned in almost all circumstances, and metaphylactic treatments (group treatments in the presence of disease) and the use of certain AMs, likely the EMA category B drugs [64], will only be allowed where clearly justified [88]. This will require significant changes in management practices on Irish farms as well as veterinary prescribing practices. One feature of prophylactic use is that it may happen whether disease is present or not. This presents an opportunity in that unnecessary use is easier to eliminate and sizeable reductions have been achieved in some countries with similar patterns of AMU, such as the United Kingdom, in recent years [89]. Reducing the need for AMU is more challenging, however, requiring improvements in overall herd health. Irish farmers previously identified economic concerns over international competition and cost effectiveness of alternatives to AMU as barriers to reducing in-feed medication [40], and there were similar findings amongst British farmers, where high investment costs and staffing difficulties were cited as barriers [30]. Nevertheless, this challenge has been met in other countries [90,91] and can be achieved without impacting performance or profit [92,93,94]. One challenge facing farmers is the forthcoming ban on the use of zinc oxide in the EU from 2022 [95]. In common with other countries such as Denmark [96], zinc oxide is widely used to control gastroenteritis on Irish farms: all but one of the study farms used it in at least one diet in 2016 (data not shown). Data on the prevalence of porcine gastrointestinal disease in Ireland are lacking and none were available for use in this study. Therefore, it is unknown how gastrointestinal disease influences AMU on Irish pig farms and future research would be of benefit. This study shows where some improvements could be made to help farms reduce AMU. Further research is needed to explore if farms practising home milling have advantages in management or nutrition that can be applied to other farms. Farms should ensure they have adequate parasite control regimens in place. High farm prevalence of pericarditis warrants a detailed investigation since there are multiple aetiologies, and indeed this is a poorly researched topic. The structure of the Irish pig industry presents some advantages in terms of AMU, many of which have not been utilised. Firstly, the sector is relatively small—there are approximately 300 commercial pig farms in Ireland. This means that potential targeted interventions to aid a reduction in AMU, for example farms with high prevalence of pericarditis, would involve a small number of farms. Secondly, almost all herds are closed, meaning that neither gilts nor fattening pigs are purchased from other farms. These practices (i.e., purchase of animals) have been associated with increased respiratory disease [97] and mortality [98]. While AMU and respiratory disease are not lower in Ireland at present, the structure of the industry may make it easier to sustain any improvements it can make.

## 5. Conclusions

This study identified prophylactic use and respiratory disease as the main drivers for antimicrobial use in Irish pig production. Farms that milled their own feed had lower AMU, while farms with higher prevalence of pericarditis, lung abscesses and liver milk spot lesions, as well as with higher finisher mortality, and those vaccinating for IAv had higher AMU. Farms that administered prophylactic AM treatments to sows or piglets had higher use of HP CIAs. These findings highlight areas of farm management where interventions may aid in reducing AMU on Irish pig farms, although further investigations at the individual farm level and in the wider population are required.

## Figures and Tables

**Table 1:** Antimicrobial use on 52 Irish farrow-to-finish pig farms during 2016 summarised by mode of treatment. The number of farms using each antimicrobial class and the mean and median treatment incidence (TI200) for each mode of treatment is shown.

Antimicrobial Class	Farms with Use	Mean (SD)	Median (Range)
*Oral group treatments*			
Tetracylines	38 (73.1%)	7.7 (12)	1.2 (0–49.1)
Potentiated sulphonamides	17 (32.7%)	6.6 (22.8)	0 (0–150.1)
Penicillins	36 (69.2%)	2.7 (5.5)	0.9 (0–29.7)
Macrolides	23 (44.2%)	2.3 (4.4)	0 (0–23.6)
Lincosamides	10 (19.2%)	0.1 (0.5)	0 (0–2.9)
Amphenicols	7 (13.5%)	0.1 (0.4)	0 (0–2.5)
Aminoglycosides	27 (51.9%)	0.5 (1.3)	0 (0–8.9)
Aminocyclitols *	10 (19.2%)	0.1 (0.3)	0 (0–1.9)
Polymyxins	13 (25%)	0.8 (2.6)	0 (0–13)
**Total oral group treatments (TI_group_)**	**50 (96.2%)**	**21 (28.5)**	**13.3 (0–167.8)**
*Individual treatments*			
Tetracyclines	22 (42.3%)	0.1 (0.2)	0 (0–0.9)
Potentiated sulphonamides	8 (15.4%)	0.1 (0.3)	0 (0–2.2)
Penicillins	52 (100%)	0.6 (0.6)	0.4 (0–2)
Macrolides	18 (34.6%)	0.2 (0.6)	0 (0–4)
Lincosamides	21 (40.4%)	0 (0)	0 (0–0.2)
Amphenicols	5 (9.6%)	0 (0)	0 (0–0.3)
Aminoglycosides	14 (26.9%)	0 (0.1)	0 (0–0.3)
Aminocyclitols	23 (44.2%)	0 (0)	0 (0–0.2)
Fluoroquinolones	45 (86.5%)	0.2 (0.3)	0.1 (0–1.2)
Cephalosporins	12 (23.1%)	0.2 (0.8)	0 (0–4.6)
Pleuromutilins	1 (1.9%)	0 (0)	0 (0–0)
**Total individual treatments (TI_individual_)**	**52 (100%)**	**1.5 (1.6)**	**1.1 (0.1–11.4)**
**Total antimicrobial use (TI_total_)**	**52 (100%)**	**22.5 (28.6)**	**14.2 (0.2–169.1)**
**Cephalopsorin and/or fluoroquinolone use (TI_ceflq_)**	**47 (90.4%)**	**0.5 (0.9)**	**0.2 (0–5.8)**

* Aminocyclitols in group treatments were used in combination with lincosamides. Legend: TI—treatment incidence.

**Table 2:** Summary of multivariable linear regression models for total antimicrobial use (AMU) (TI_total_), group oral AMU (TI_group_), individual AMU (TI_individual_) and cephalosporin/fluoroquinolone use (TI_ceflq_). The outcome variables are expressed as treatment incidence (TI200) and were log-transformed prior to analysis. Both the coefficients on the log scale and the back-transformed estimates are presented.

Outcome Variable	Predictor Variables	Estimate ^a^	Std. Error	Back-Transformed Estimate (95% CI)	*p*
LOG TI_total_	Intercept	3.28	0.936	26.48	
adjusted R^2^ = 0.53	Finisher mortality_2016	0.28	0.135	1.32 (1.01–1.74)	0.043
*p* < 0.001	Home milling (ref = no)	−1.08	0.265	0.34 (0.2–0.58)	0.000
	Weaner stage (days)	−0.03	0.013	0.97 (0.95–1)	0.027
	IAv vaccination (ref = no)	1.06	0.272	2.88 (1.66–4.97)	0.000
	Lung abscesses (%)	0.07	0.037	1.07 (0.99–1.16)	0.070
	Pericarditis (%)	0.06	0.031	1.06 (1–1.13)	0.059
LOG TI_group_	Intercept	2.93	0.832	18.72	
adjusted R^2^ = 0.55	Finisher mortality	0.28	0.120	1.33 (1.04–1.69)	0.023
*p* < 0.001	Home milling (ref = no)	−0.97	0.235	0.38 (0.23–0.61)	0.000
	Weaner stage (days)	−0.02	0.011	0.98 (0.95–1)	0.041
	IAv vaccination (ref = no)	0.95	0.242	2.58 (1.58–4.19)	0.000
	Lung abscesses (%)	0.07	0.033	1.07 (1–1.14)	0.046
	Pericarditis (%)	0.06	0.028	1.06 (1–1.12)	0.037
LOG TI_individual_	Intercept	−0.58	0.473	0.56	
adjusted R^2^ = 0.29	Feed, water and equipment supply ^b^	−0.01	0.007	0.99 (0.97–1)	0.048
*p* < 0.001	Vermin and bird control ^b^	0.01	0.005	1.01 (1–1.02)	0.018
	Piglet prophylaxis (ref = no) ^c^	0.60	0.192	1.82 (1.23–2.67)	0.003
	Liver milk spots (%)	0.01	0.004	1.01 (1–1.02)	0.018
LOG TI_ceflq_	Intercept	−1.32	0.816	0.27	
adjusted R^2^ = 0.37	Weaner mortality	−0.41	0.126	0.66 (0.51–0.85)	0.002
*p* < 0.001	Feed, water and equipment supply ^b^	−0.03	0.012	0.97 (0.95–1)	0.031
	Sow prophylaxis (ref = no) ^c^	0.72	0.378	2.06 (0.96–4.41)	
	Piglet prophylaxis (ref = no) ^c^	1.16	0.350	3.19 (1.58–6.45)	0.002
	Pericarditis (%)	0.14	0.041	1.15 (1.06–1.25)	0.001

Constants were added to the TI_individual_ and TI_ceflq_ (1 and 0.01, respectively) before log transformation. The back-transformed estimates refer to the multiplicative effect per unit change of predictor variable. For example, farms vaccinating against IAv had 2.88 times higher treatment incidence than farms not vaccinating. ^a^ Log scale. ^b^ Biocheck.Ugent^TM^ biosecurity score. ^c^ Prophylactic antimicrobial use. Legend: CI—confidence interval; TI—treatment incidence; IAv—influenza A virus.

**Table 3:** Summary of multivariable logistic regression models investigating risk factors for oral group antimicrobial treatment with various antimicrobial classes on Irish farrow-to-finish pig farms (*n* = 52) during 2016. Predictor variables with *p* ≤ 0.1 were retained in the final models. Results are presented as odds ratios with 95% confidence intervals.

	Outcome Variables: Oral Group Treatment with Antimicrobial Class							
	Tetracyclines	Potentiated Sulphonamides	Penicillins	Macrolides	Lincosamides	Amphenicols	Aminoglycosides	Polymyxins
Predictor Variable	OR (95% CI)	OR (95% CI)	OR (95% CI)	OR (95% CI)	OR (95% CI)	OR (95% CI)	OR (95% CI)	OR (95% CI)
*Herd characteristics*								
Home milling (ref = no)	0.16 (0.02–0.86)	-	-	-	-	-	-	-
Finisher mortality (%)	-	7.7 (1.7–88.88)	-	2.81 (1.32–7.18)	-	-	-	-
Age at weaning (days)	-	-	-	1.32 (1.05–1.78)	-	-	-	-
Weaner stage (days)	0.89 (0.78–0.98)	0.69 (0.45–0.87)	-	-	0.9 (0.8–0.98)	-	-	-
Finisher stage (days)	-	-	-	-	-	-	1.07 (1–1.16)	-
No. of sows per employee	-	-	-	-	-	-	0.98 (0.95–1.00)	1.02 (1–1.04)
Farmer experience (years)	-	-	-	-	-	-	1.08 (1.00–1.18)	-
Youngest building (years)	-	-	-	-	1.3 (1.07–1.68)	-	-	-
*Biosecurity scores*								
Feed, water and equipment supply	-	0.92 (0.82–1.00)	-	-	-	-	-	-
Environment and region	-	0.95 (0.87–1.00)	-	-	-	-	-	-
Nursery unit management	0.93 (0.87–0.99)	-	-	-	-	-	-	-
Measures between compartments and use of equipment	-	-	-	-	-	-	-	0.94 (0.89–0.99)
*Pluck lesions at slaughter and disease status*								
Pleurisy (%)	-	1.11 (1.03–1.25)	-	-	-	-	-	-
Moderate/severe pleurisy (%)	-	-	-	-	-	-	0.89 (0.81–0.96)	-
Scars (%)	1.13 (1.02–1.3)	-	-	-	-	-	1.1 (1.02–1.23)	-
Lung abscesses (%)	-	-	-	-	-	1.47 (1.09–2.9)	-	-
Pericarditis (%)	-	-	1.35 (1.11–1.73)	-	-	-	-	1.19 (1.01–1.44)
Liver milk spots (%)	-	-	-	-	-	1.04 (1–1.11)	-	-
IAv status (ref = negative)	-	-	0.11 (0.01–0.74)	-	-	-	-	-
Mhyo status (ref = negative)	-	-	-	-	134.31 (4.66–>9999)	-	-	-
PRRSv status (ref = negative)	-	-	-	-	-	270.67 (5.26–>9999)	-	-
*Vaccination and prophylactic antimicrobial use practices*								
IAv vaccination (ref = no)	12.53 (1.72–183.73)	106.48 (5.14–>9999)	-	24.11 (4.8–191.38)	-	-	-	-
Piglet prophylaxis (ref = no)	-	-	10.18 (2.22–64.81)	-	-	11.3 (1.09–367.08)	-	-

To aid readability of the table, *p*-values are omitted. A full version of the table including *p*-values is available in the Supplementary Material, Table S7. Legend: OR-odds ratio; CI-confidence interval; IAv—influenza A virus; Mhyo—*Mycoplasma hyopneumoniae*; PRRSv—porcine respiratory and reproductive syndrome virus.

**Table 4:** Summary of multivariable logistic regression models investigating risk factors for individual antimicrobial treatment with various antimicrobial classes on Irish farrow-to-finish pig farms (*n* = 52) during 2016. Results are presented as odds ratios with 95% confidence intervals.

	Outcome Variables: Individual Treatment with Antimicrobial Class							
	Tetracyclines	Potentiated Sulphonamides	Macrolides	Lincosamides	Aminoglycosides	Aminocyclitols	Fluoroquinolones	Cephalosporins
Predictor variable	OR (95% CI)	OR (95% CI)	OR (95% CI)	OR (95% CI)	OR (95% CI)	OR (95% CI)	OR (95% CI)	OR (95% CI)
*Herd characteristics*								
Piglet mortality (%)	-	-	0.77 (0.57–0.98)	-	-	-	-	-
Weaner mortality (%)	1.78 (1.04–3.66)	-	-	-	-	-	-	-
Finisher mortality (%)	-	-	0.52 (0.22–1.04)	-	-	-	-	-
Average daily gain (g)	-	-	-	1.02 (1–1.03)	-	-	-	0.99 (0.97–1)
Feed conversion ratio	0 (0–0.15)	-	-	-	-	-	-	-
Pigs per sow per year	-	-	-	-	0.43 (0.2–0.73)	0.61 (0.38–0.89)	-	-
Weaning age (days)	-	0.72 (0.5–0.95)	-	-	0.79 (0.6–0.97)	-	-	-
Total rearing period (days)	-	-	-	-	-	0.91 (0.83–0.97)	-	-
No. of sows per employee	-	-	-	-	1.04 (1.01–1.07)	-	-	1.03 (1.01–1.06)
*Biosecurity scores*								
Transport of animals, removal of manure and dead animals	-	-	1.11 (1.03–1.23)	-	-	-	-	-
Feed, water and equipment supply	-	-	-	-	-	-	0.9 (0.8–0.97)	-
Vermin and bird control	-	1.08 (1.02–1.19)	-	-	-	-	-	-
Disease management	0.97 (0.93–1.00)	-	-	-	-	-	-	-
Fattening unit management	1.06 (1.01–1.13)	1.07 (1.01–1.16)	-	-	-	-	-	-
Overall internal biosecurity	-	-	-	-	-	1.06 (1.01–1.13)	-	-
*Pluck lesions at slaughter and disease status*								
Pericarditis (%)	-	-	-	-	-	0.82 (0.65–0.98)	1.59 (1.11–2.67)	-
Liver milk spots (%)	0.96 (0.93–0.99)	-	-	1.04 (1.01–1.07)	-	-	-	-
IAv status (ref = negative)	-	-	-	-	0.07 (0–0.51)	-	-	-
*Vaccination and prophylactic antimicrobial use practices*								
IAv vaccination (ref = no)	-	-	-	8.18 (1.8–50.29)	-	-	-	-
PRRSv vaccination (ref = no)	-	-	-	7.27 (1.54–45.87)	-	-	-	-
Piglet prophylaxis (ref = no)	-	0.01 (0–0.21)	-	-	-	-	-	4.35 (0.96–26.2)
Sow prophylaxis (ref = no)	-	-	-	-	-	-	17.22 (1.15–1032.14)	-

To aid readability of the table, *p*-values are omitted. A full version of the table including *p*-values is available in the Supplementary Material, Table S8. Legend: OR-odds ratio; CI-confidence interval; IAv—influenza A virus; PRRSv—porcine respiratory and reproductive syndrome virus.

## Data Availability

The datasets used and/or analysed during the current study are available from the corresponding author upon reasonable request.

## Supplementary Materials

The following are available online at https://www.mdpi.com/article/10.3390/ani11102828/s1, Figure S1: Venn diagram summarising co-infection with four respiratory pathogens on 52 Irish farrow-to-finish pig farms. Table S1: Summary of farm characteristics and performance for 52 Irish farrow-to-finish pig farms from 2016. Table S2: Summary of biosecurity scores evaluated using the Biocheck.UGent™ questionnaire from 52 Irish farrow-to-finish pig farms in 2016. Table S3: Summary of prevalence of pluck lesions and disease status on 52 Irish farrow-to-finish pig farms. Table S4: Summary of vaccination and prophylactic antimicrobial use practices on 52 Irish farrow-to-finish pig farms during 2016. Table S5: Summary of *p*-values for the univariable linear regression models investigating risk factors for antimicrobial use (AMU) on 52 Irish farrow-to-finish pig farms during 2016. Table S6: Summary of univariable associations between a range of risk factors and the use of various antimicrobial classes via oral group or individual modes of treatment on 52 Irish farrow-to-finish pig farms during 2016. Table S7: Summary of multivariable logistic regression models investigating risk factors for oral group antimicrobial treatment with various antimicrobial classes on Irish farrow-to-finish pig farms (*n* = 52) during 2016 (this is a companion to Table 3). Table S8: Summary of multivariable logistic regression models investigating risk factors for oral group antimicrobial treatment with various antimicrobial classes on Irish farrow-to-finish pig farms (*n* = 52) during 2016 (this is a companion to Table 4).
