# Supplementary material for: Risk Factors for Antimicrobial Use on Irish Pig Farms
Source: Animals (Basel). 2021 Sep 28;11(10):2828. doi: 10.3390/ani11102828 (PMC8532697; doi:10.3390/ani11102828)
Supplement: Supplementary file 1 [file animals-11-02828-s001.zip › Figure S1.pdf]

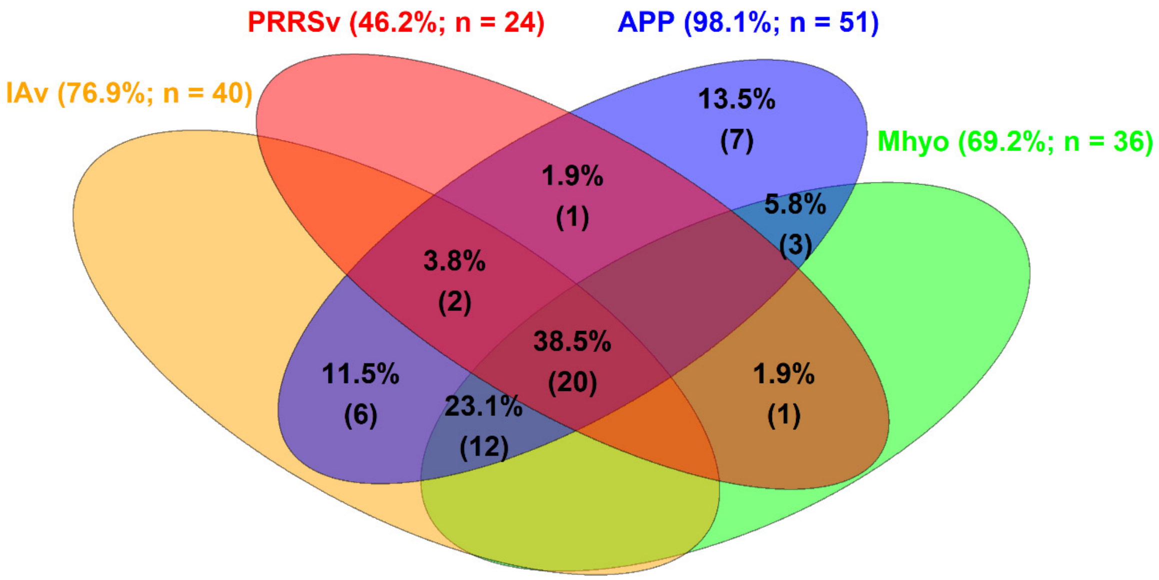

Figure S1. Venn diagram summarising co-infection with four respiratory pathogens on 52 Irish farrow-to-finish pig farms. Disease status for each farm was determined using serological testing.

Legend: IAv - influenza A virus, PRRSv - porcine respiraotory and reproductive syndrome virus; APP - *Actinobacillus pleuropneuomiae*; Mhyo - *Mycoplasma hyopneumoniae*
