# Supplementary material for: Risk Factors for Antimicrobial Use on Irish Pig Farms
Source: Animals (Basel). 2021 Sep 28;11(10):2828. doi: 10.3390/ani11102828 (PMC8532697; doi:10.3390/ani11102828)
Supplement: Supplementary file 1 [file animals-11-02828-s001.zip › Tables S1 - S5.pdf]

Table S1. Summary of farm characteristics and performance for 52 Irish farrow-to-finish pig farms in 2016.

	**mean (SD)**	**median (range)**
Herd size (no. of sows)	745.5 (511.8)	650 (113 - 2354)
Piglet mortality (%)	9.9 (2.8)	9.7 (1.3 - 16.3)
Weaner mortality (%)	2.6 (1.5)	2.4 (0.7 - 9.4)
Finisher mortality (%)	2.2 (1.0)	2 (0.8 - 5.1)
Average daily gain (g)	704.4 (60.2)	699.5 (554 - 856)
Feed conversion ratio	2.4 (0.1)	2.4 (2.1 - 2.8)
Pigs produced per sow per year	26.1 (2)	26 (22.4 - 31)
Age at weaning (days)	29.3 (3.8)	29 (19 - 37)
Weaner stage (days)	66 (10.1)	66.6 (38.3 - 94.1)
Finisher stage (days)	76.7 (10.6)	75.8 (60.7 - 102.2)
Total rearing period (days)	172 (12.6)	169.5 (146 - 210)
No. of sows per employee	157.9 (39.5)	160.5 (56.5 - 237.4)
Farmer experience (years)	27 (10.2)	30 (5 - 50)
Age of youngest building (years)	3.7 (5.0)	2.5 (0 - 25)
Age of oldest building (years)	36.1 (26.5)	33.5 (12 - 210)

Table S2. Summary of biosecurity scores evaluated using the Biocheck.UGent™ questionnaire from 52 Irish farrow-to-finish pig farms in 2016.

**Category**	**mean (SD)**	**median (range)**
External biosecurity		
Purchase of animals and semen	98.8 (5.2)	100 (70 - 100)
Transport of animals, removal of manure and dead animals	80.4 (11.2)	83 (43 - 96)
Feed, water and equipment supply	54.6 (15.1)	53 (10 - 80)
Personnel and visitors	74.1 (18.8)	76 (24 - 100)
Vermin and bird control	68.7 (20)	70 (30 - 100)
Environment and region	83.1 (21.6)	90 (20 - 100)
Overall external biosecurity score	79.2 (7.9)	79.5 (62 - 94)
Internal biosecurity		
Disease management	82.3 (22.6)	100 (20 - 100)
Farrowing and suckling period	53 (19.5)	53.5 (7 - 86)
Nursery unit management	63.2 (16.3)	67.5 (36 - 100)
Finisher unit management	73.2 (22)	79 (21 - 93)
Measures between compartments and the use of equipment	50.1 (16.1)	50 (21 - 86)
Cleaning and disinfection	41.2 (27.9)	38 (0 - 95)
Overall internal biosecurity score	57.2 (14.4)	60 (29 - 80)
Overall biosecurity score	68.4 (9.7)	70 (47 - 87)

Table S3. Summary of vaccination and prophylactic antimicrobial use practices on 52 Irish farrow-to finish pig farms during 2016.

	Response	
	Yes	No
	n (%)	n (%)
Vaccination practices (yes vs no)		
Swine influenza virus	19 (36.5%)	33 (63.5%)
PRRSv	21 (40.4%)	31 (59.6%)
Porcine parvovirus	52 (100%)	0 (0%)
Erysipelothrix rhusiopathiae	52 (100%	0 (0%)
PCV2	49 (94.2%)	3 (5.8%)
Mycoplasma hyopneumoniae	39 (75.0%)	13 (25%)
Escherichia coli	44 (84.6%)	8 (15.4%)
Glaesserella parasuis	0 (0%)	52 (100%)
Actinobacillus pleuropneumoniae	5 (9.6%)	47 (90.4%)
Clostridium species	9 (17.3%)	43 (82.7%)
Atrophic rhinitis	4 (7.7%)	48 (92.3%)
Prophylactic use of antimicrobials in stage (yes vs no)[a]		
Sow	20 (38.5%)	32 (61.5%
Piglet	26 (50%)	26 (50%)
Weaner	7 (13.5%)	45 (86.5%)
Finisher	2 (3.8%)	50 (86.2%)

[a] Does not include administration of medicated feed to piglets, weaners, or finishers

Legend: PRRSv - porcine reproductive and respiratory syndrome virus; PCV2 - porcine circovirus 2

Table S4. Summary of prevalence of pluck lesions and disease status on 52 Irish farrow-to-finish pig farms. Disease status was determined based on serology. Evaluation of pluck lesions and collection of blood for serology were carried out at slaughter.

**Lesion**	**mean (SD)**	**median (range)**
Pleurisy	13.8 (15.2)	7.2 (0.4 - 51.8)
Moderate to severe pleurisy	11.5 (12.8)	6.2 (0 - 46.9)
Cranial pleurisy	15.5 (13.4)	10.8 (1.2 - 54.2)
Pneumonia	10.6 (10.9)	7.7 (0 - 50)
Scars	13.2 (9.7)	11.1 (0 - 39.3)
Lung abscesses	1.3 (3.5)	0.2 (0 - 21.5)
Pericarditis	8.1 (4.4)	7.6 (1.2 - 19.1)
Liver milk spots	28.6 (27.3)	17.9 (0.4 - 97.3)
**Disease status**	**positive**	**negative**
Actinobacillus pleuropneumoniae	51 (98.1%)	1 (1.9%)
PRRSv	24 (46.2%)	28 (53.8%)
Swine influenza virus (IAv)	40 (76.9%)	12 (23.1%)
Mycoplasma hyopneumoniae	36 (69.2%)	16 (30.8%)

Legend: IAv - influenza A virus; PRRSv - porcine reproductive and respiratory syndrome virus; Mhyo - *Mycoplasma hyopneumoniae*

Table S5. Summary of P-values for the univariable linear regression models investigating risk factors for antimicrobial use (AMU) on 52 Irish farrow-to-finish pig farms during 2016. The outcome variables were total AMU [TI_{total}], group oral AMU (TI_{group}), individual AMU ($TI_{individual}$) and cephalosporin/fluoroquinolone use (TI_{ceflq}) expressed as Treatment Incidence (TI200). The outcome variables were log transformed prior to analysis.

	**Outcome variables**			
Predictor variables	**TI_{total}**	**TI_{group}**	**$TI_{individual}$**	**TI_{ceflq}**
Herd characteristics				
Herd size (no. of sows)	0.425	0.367	0.694	0.911
Piglet mortality (%)	0.947	0.865	0.389	0.562
Weaner mortality (%)	0.828	0.835	0.404	0.225
Finisher mortality (%)	0.003	0.002	0.78	0.7
Average daily gain (g)	0.622	0.741	0.725	0.683
Feed conversion ratio	0.613	0.542	0.657	0.46
Pigs produced per sow per year	0.894	0.942	0.615	0.361
Age at weaning (days)	0.204	0.12	0.354	0.228
Weaner stage (days)	0.153	0.204	0.472	0.304
Finisher stage (days)	0.197	0.151	0.747	0.632
Total rearing period (days)	0.75	0.519	0.26	0.954
No. of sows per employee	0.952	0.887	0.828	0.451
Farmer experience (years)	0.789	0.857	0.705	0.84
Age of youngest building (years)	0.943	0.918	0.355	0.896
Age of oldest building (years)	0.736	0.737	0.805	0.504
Home milling (*ref = no*)	0.001	0.001	0.747	0.784
Biosecurity scores				
Purchase of animals and semen	0.975	0.906	0.434	0.618
Transport of animals, removal of manure and dead animals	0.867	0.998	0.55	0.302
Feed, water and equipment supply	0.057	0.133	0.147	0.065
Personnel and visitors	0.462	0.691	0.332	0.814
Vermin and bird control	0.802	0.857	0.212	0.624
Environment and region	0.892	0.679	0.79	0.656
Overall external biosecurity score	0.335	0.58	0.362	0.512
Disease management	0.255	0.159	0.975	0.831
Farrowing and suckling period	0.26	0.365	0.632	0.694
Nursery unit management	0.61	0.652	0.86	0.459
Finisher unit management	0.919	0.979	0.676	0.594
Measures between compartments and the use of equipment	0.461	0.64	0.989	0.995
Cleaning and disinfection	0.478	0.617	0.463	0.161
Overall internal biosecurity score	0.313	0.42	0.62	0.406
Overall biosecurity score	0.254	0.411	0.468	0.392

Legend: IAv - influenza A virus; PRRSv - porcine reproductive and respiratory syndrome virus; Mhyo - *Mycoplasma hyopneumoniae*

Table S5. continued

Predictor variables	Outcome variables			
	TI_{total}	TI_{group}	$TI_{individual}$	TI_{ceflq}
Pluck lesions at slaughter and disease status				
Pleurisy (%)	0.05	0.035	0.959	0.392
Moderate to severe pleurisy (%)	0.049	0.033	0.983	0.352
Cranial pleurisy (%)	0.045	0.032	0.895	0.366
Pneumonia (%)	0.562	0.707	0.382	0.031
Scars (%)	0.098	0.091	0.879	0.51
Lung abscesses (%)	0.029	0.02	0.322	0.986
Pericarditis (%)	0.005	0.003	0.928	0.026
Liver milk spots (%)	0.712	0.427	0.046	0.383
PRRSv status (*ref = no*)	0.869	0.911	0.968	0.162
Swine influenza virus (IAv) status (*ref = no*)	0.286	0.335	0.75	0.755
Mhyo status *(ref = no)*	0.957	0.994	0.159	0.759
Vaccination and prophylactic antimicrobial use practices				
Piglet prophylaxis (*ref = no*)	0.134	0.17	0.246	0.197
Sow prophylaxis (*ref = no*)	0.613	0.969	0.002	0.007
IAv vaccination (*ref = no*)	0.009	0.009	0.237	0.266
PRRSv vaccination *(ref = no)*	0.352	0.405	0.838	0.875
Mhyo vaccination (*ref = no*)	0.118	0.189	0.963	0.92

Legend: IAv - influenza A virus; PRRSv - porcine reproductive and respiratory syndrome virus; Mhyo - *Mycoplasma hyopneumoniae*
